# Supplementary material for: Progesterone Dampens Immune Responses in In Vitro Activated CD4+ T Cells and Affects Genes Associated With Autoimmune Diseases That Improve During Pregnancy
Source: Front Immunol. 2021 May 12;12:672168. doi: 10.3389/fimmu.2021.672168 (PMC8149943; doi:10.3389/fimmu.2021.672168)
Supplement: Supplementary file 1 [file DataSheet_1.docx]

**Supplementary information**

**
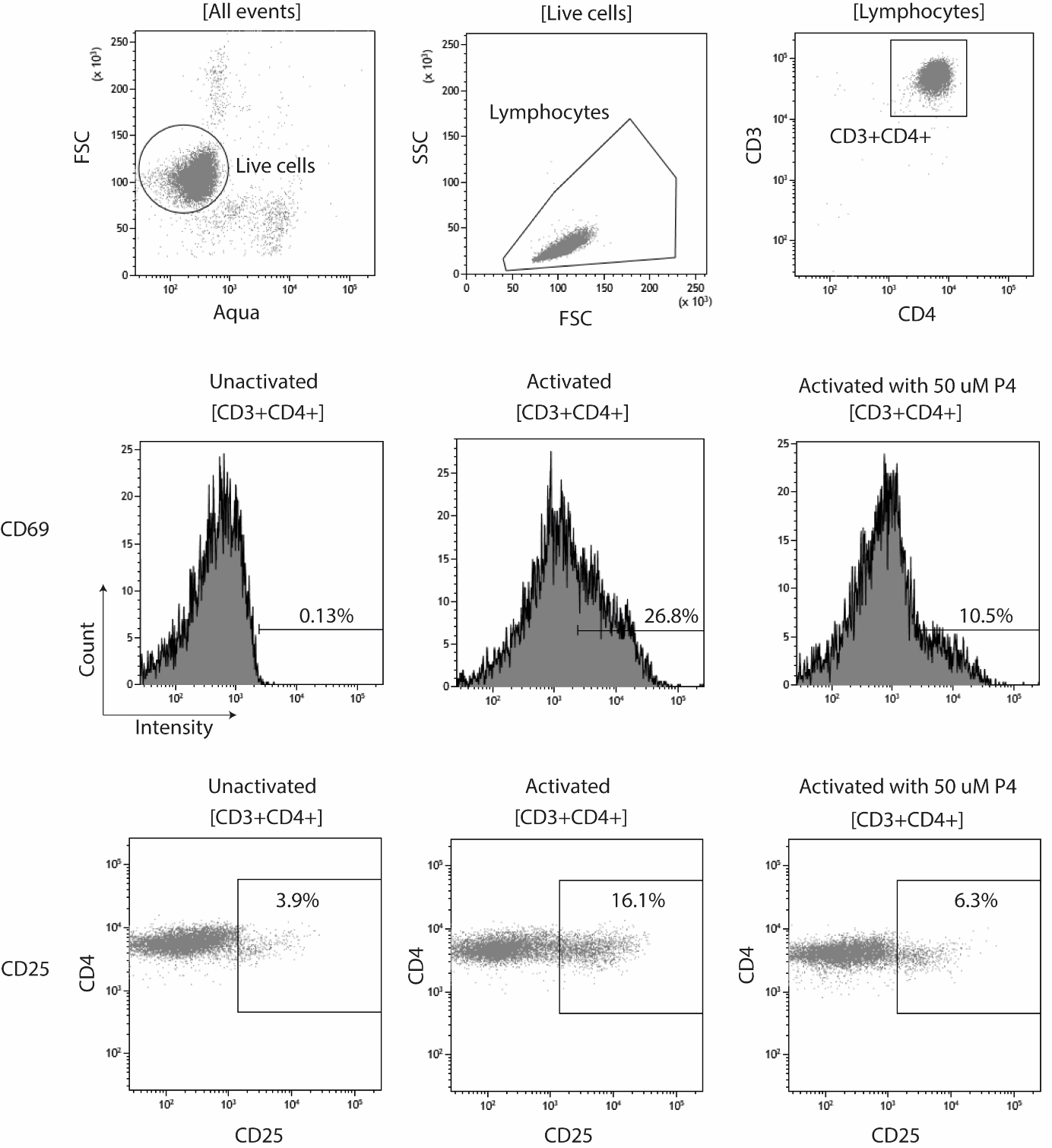
**

**Figure S1.** Gating strategy for analysis of T cell activation markers. Cells were gated as live cells based on negative expression of Aqua Live/Dead stain and gated based on forward and side scatter. CD3^+^CD4^+^ T cells were analysed for the expression of CD69 (6 and 24 hrs) and CD25 (24 hrs) and the percentage of cells expression the markers after activation was set based on the expression in the unactivated cells. Figure shows one representative sample. FSC: forward scatter, P4: progesterone, SSC: side scatter.


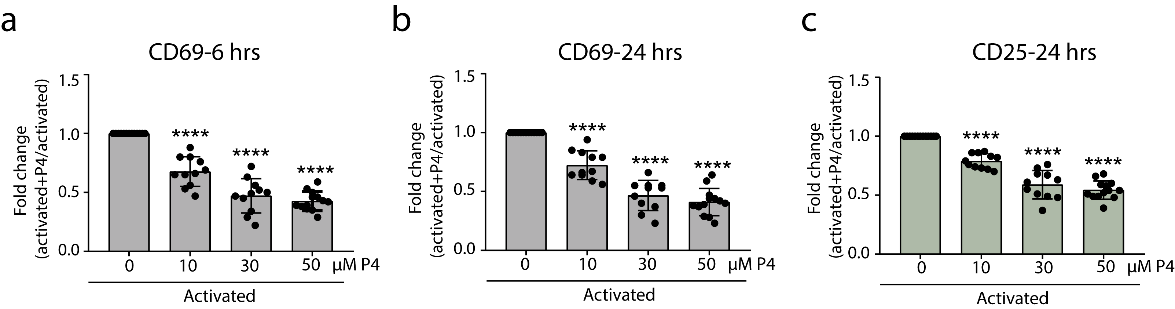


**Figure S2.** Fold change in activation markers in CD4^+^ T cells activated in the presence of P4. Isolated CD4^+^ T cells were activated in the presence or absence of 50 µM of P4 for 6 and 24 hrs. Activation status was evaluated by flow cytometry using the surface activation markers ( **(A-B)** CD69 (6 and 24 hrs) and **(C)** CD25 (24 hrs). Figure shows mean ± standard deviations of the fold-change in expression of the activation markers comparing activation in the presence of P4 as compared to activation alone. Statistical differences were determined using one-way ANOVA with Dunnett’s multiple comparison test. n=13, ****p≤0.0001. P4: progesterone.
